# Synthesis of Nanoparticles of Different Morphology in a DC Discharge

**DOI:** 10.3390/nano15231802

**Published:** 2025-11-29

**Authors:** Alexander V. Fedoseev, Anatoly V. Filippov, Mikhail M. Vasiliev, Oleg F. Petrov

**Affiliations:** Joint Institute for High Temperatures of the Russian Academy of Sciences, Moscow 125412, Russia; fav@triniti.ru (A.V.F.); vasiliev@ihed.ras.ru (M.M.V.);

**Keywords:** nanoparticles synthesis, stratified DC glow discharge, material sputtering, plasma-particles interaction

## Abstract

The synthesis of nanoparticles in the plasma of a stratified DC gas discharge was carried out under typical gas discharge conditions, i.e., at room temperature and an argon pressure of 0.11 torr, with a discharge current of 2.5 mA. The particles were formed and grown due to the sputtering of a dielectric plasma concentrator, which was used for strata stabilization. The analysis of the material collected using double-sided carbon tape placed on the glass wall of the discharge tube was performed by scanning electron microscopy and X-ray energy-dispersive microanalysis after the experiments. Three distinctive groups of particles of different shape and size were found, i.e., smooth spherical nanoparticles with a size of 10–100 nm, the main group of smooth spherical and dumbbell-like particles with a size of 200–500 nm, and micron-sized particles of complex cauliflower-like shape. EDX microanalysis of the synthesized nanoparticles revealed that the particles mainly consist of C, O, and Si, which proves that they were formed from the sputtered material of the silicone dielectric concentrator. Analysis of the particles and plasma parameters was performed, and a probable mechanism for the formation of such particles is proposed.

## 1. Introduction

The possibility of the formation of solid particles and their conglomerates in a discharge plasma has been known for a long time [1]. Spontaneous uncontrolled growth of particles ranging in size from several nanometers to several micrometers can occur directly in the bulk of the discharge plasma in the processes of coagulation of the working gas dissociation products [2] or due to sputtering of substrate material into the plasma [3]. Such large particles compared to electrons, ions, gas atoms, and molecules are often referred to as dust particles, as they lead to undesirable contamination of products obtained in microelectronics. The formation of dust particles was observed in the plasma of discharges of various types: rf discharges [2,3,4,5,6], dc discharges [7,8,9,10,11,12,13], arc discharges [1,14], microwave discharges [15], magnetron plasma [16], in fusion devices [17], as well as in space [18].

To date, the formation of dust particles in the plasma of RF discharges in monosilane used in microelectronics is one of the most studied [2,3,4,5,19,20]. To understand where the solid particles come from and pollute thin films produced in the semiconductor industry, the interelectrode discharge gap was illuminated with a laser [2,3,4,5]. Since the reflected light from the surfaces of micron-sized solid particles is easily visible even with the naked eye, it was found that the particles form and levitate directly in the discharge in the electric potential of the substrate. The ease of micron-sized particle observation with a laser has led to the rapid development of a new area of plasma physics, the cold dusty plasma or plasma with the disperse phase (see, e.g., reviews [21,22]). In laboratory conditions, the calibrated particles of a given micrometer size are introduced into the discharge plasma for the investigation of various physical processes of their interaction. Such particles are charged to large (often negative) charges, are captured in the discharge plasma under the action of near-electrode and near-wall electromagnetic fields, and are observed in the form of clouds of dust particles. Note that the particles themselves are repelled from each other by means of the Coulomb screened interaction potential. The Coulomb coupling parameter Γ can reach large values in laboratory plasma when the potential energy of their interaction exceeds their kinetic energy by a couple of hundred times [23]. Therefore, many different phenomena characteristic of nonideal Coulomb systems are observed in the subsystem of dust particles, including the formation of dust crystals [23,24,25], phase transitions [25,26], dust-acoustic waves [11,27], and many others. Moreover, dust particles can be considered not only as unwanted pollutants, but they have potentially useful properties, e.g., very small sizes (nanometer to micrometer range), uniform size distribution, and chemical activity [28].

Along with RF discharges, there is a fairly large number of works on the synthesis of nanoparticles in the plasma of DC discharges [7,8,9,10,11,12,13]. In [7], the processes of formation and growth of nanoparticles were studied in a cylindrical discharge tube in a mixture of argon and acetylene on the so-called Plasma Kristall-4 setup. Formation and dynamics of dust nano and micro-particles in a spherical stratified DC discharge in high molecular gases (ethanol, acetylene) were presented in [8,9,10]. In [9], the presence of elongated particles about 2 mm long, which were oriented along the radial component of the electric field, was found. Sputtering of the cathode in [10] led to the formation of complex nanosized clusters, which present an iron core encapsulated by a carbon nanomaterial.

Recently, the synthesis of nanoparticles up to 100 nm in size was carried out in an ultra-cold plasma (*T* ≈ 2 K) of a stratified DC discharge cooled by superfluid helium [11,12,13]. It was assumed that nanoparticles are formed as a result of the condensation of the sputtering material of a conical clay inserted into the discharge tube due to bombardment by electron and ion beams. Fast growth of quasi one-dimensional structures and filaments with a high aspect ratio of 100 in ultracold plasma was explained by self-assembly of polarizable materials in strongly inhomogeneous electric fields. The key parameters of such a synthesis are low gas temperature (*T* ≈ 2 K) and low released power (0.1 W) in the discharge. It should be noted that the process of formation and growth of nanoparticles in such a discharge at room temperature has not yet been observed.

In this work, the synthesis of nano- and micro-particles in the plasma of a stratified DC glow discharge at room temperature is carried out for the first time. Three distinctive groups of particles of various shapes and sizes were found, namely, small spherical nanoparticles with a size of 10–100 nm, the main group of particles of smooth spherical shape with a size of 200–500 nm, and micron-sized particles of complex shape. The paper presents a description of an experimental setup for the synthesis of the particles, the results obtained with SEM images of the resulting nano- and micro-particles, EDX microanalysis of the particle material, and an analysis of possible mechanisms of the formation of such groups of particles.

## 2. Experimental Setup and Results

**Figure 1:**
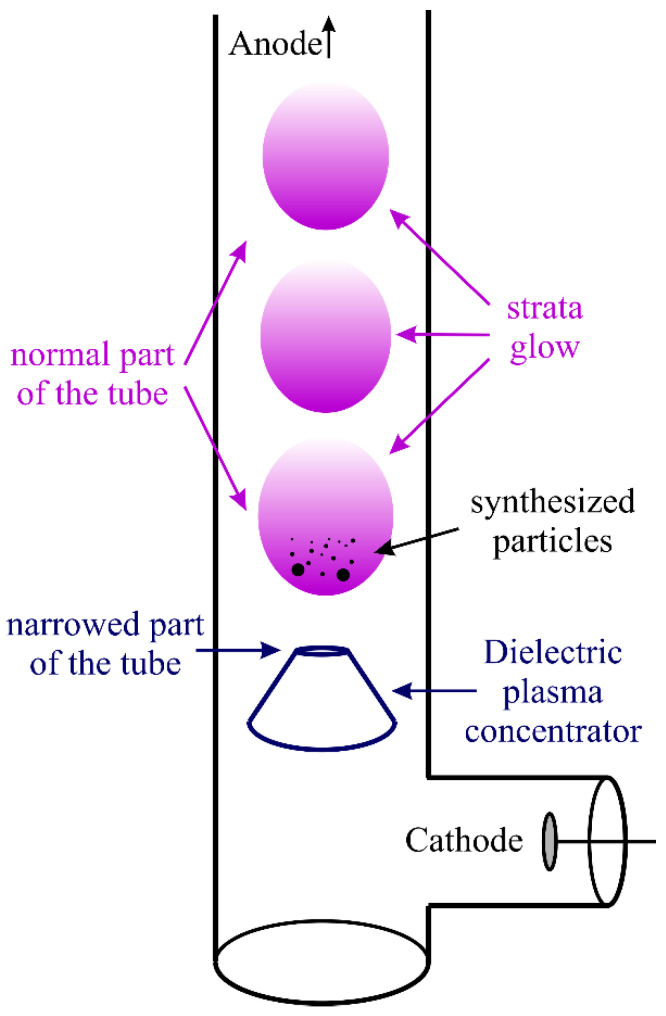
Experimental setup.

The experimental setup consists of a vertically oriented glass gas-discharge tube and two side branches with electrodes (see Figure 1). The lower branch houses the cathode, while the upper branch houses the anode. A stainless steel electrode was used as the cathode, which prevented (or reduced) the formation of an oxide film, which significantly impacts the stability of discharge generation. Experiments with cathodes made of other materials showed unstable discharge generation after some time of use; thus, they were not used in these experiments. Gas pumping and inlet systems are connected to the upper part of the tube via a vacuum flange. Argon was used as the plasma-forming gas. The vacuum system consists of a series-connected fore-vacuum and turbomolecular pumps, as well as shutoff valves and a gas supply module, controlled by an electronic programmable pressure control unit. A special dielectric plasma concentrator made of polydimethylsiloxane (PDMS) [Si(CH_3_)_2_O]*n* is located at the bottom part of the gas discharge tube (see Figure 1). One of the functions of this concentrator is the positioning of the lower stratum and stabilization of the stratified discharge in the tube. Furthermore, this dielectric concentrator can be used for the synthesis of nano- and micro-particles in the gas phase due to sputtering of its material by the discharge current.

A solid-state laser with a wavelength of 532 nm was used to illuminate the cloud of nanoparticles. The power of 0.5 W was selected to minimize the impact on the particles while still being sufficient for illumination (observation) of the particles. The beam diameter was approximately 30 mm to illuminate the entire discharge region where the particle cloud is forming. It was directed axially into the discharge tube through its flat lower end.

First, the gas discharge tube was pumped out using the described above vacuum system to a residual pressure below 10^−5^ Pa. Then, the working gas (argon) injection system was turned on to reach a pressure of 14.7 Pa. An electric potential difference of 3 kV was established between the cathode and anode, which caused electrical breakdown and the formation of a DC glow discharge. The discharge current was set to be 2.5 mA. It is worth noting that as the dielectric concentrator was sputtered, levitating microparticles of submicron and micron sizes were observed in the first stratum above the concentrator.

**Figure 2:**
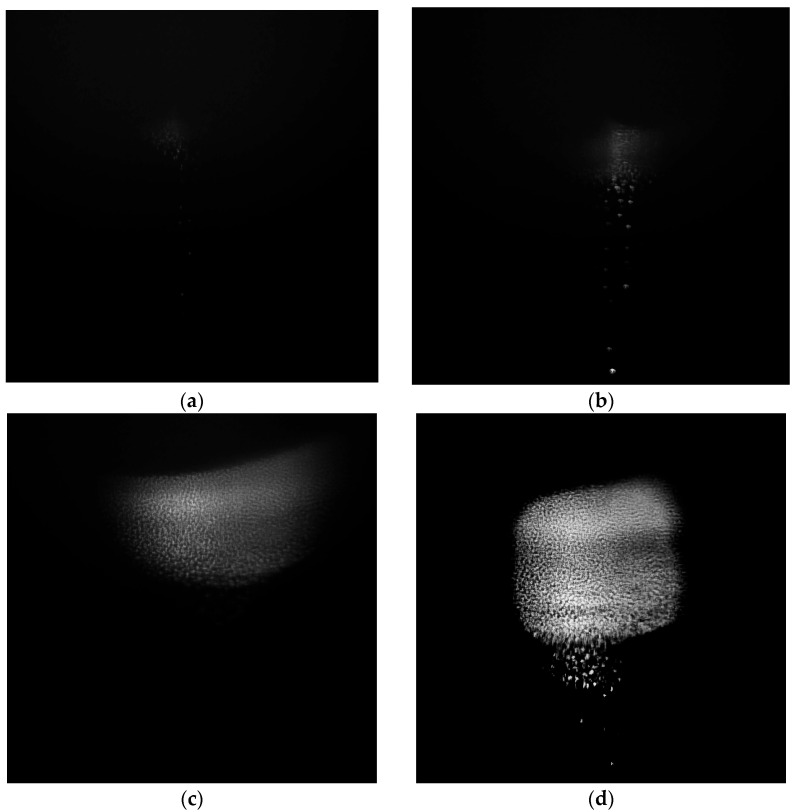
Video frames of 10 × 10 mm in size with the same exposure of the particle structure at different times after the discharge is turned on: (**a**) 2 min; (**b**) 5 min; (**c**) 7 min; (**d**) 10 min.

Temporal evolution of the particle formation and growth in a glow discharge was as follows. Initially, during sputtering of the dielectric concentrator, the formation of a condensed dispersed phase was observed in the first stratum of the stratified glow discharge above the concentrator (see Figure 1 and Figure 2). The video frames shown in Figure 2 were obtained with a high-speed camera IDT XStream 1440p (USA) with a maximum image resolution of 2560 × 1440 pixels and sensor pixels of 7.00 × 7.00 µm. The formation of the submicron fraction was visually observed as a “haze”, in which individual particles were initially indistinguishable (see Figure 2a). After 2, 5, 7, and 10 min from the onset of plasma generation and the concentrator sputtering, an increase in the number and size of the particles was observed (see Figure 2b–d). It should be noted that these videoframes were obtained with the same exposure, and the intensity of the particles in the photographs determines their size.

It should be noted that the particles are illuminated by coherent laser radiation; thus, the obtained video frames are not actual particle images, but rather scattering spots. The size of these spots depends on the scattering indicatrix (particle shape, size, scattering angle, particle material, etc.). It is impossible to directly determine the exact particle size from the experimental video recordings. Only the particle positions (coordinates) and, consequently, the interparticle distances can be reliably determined. The growth of the particle size can be indirectly assessed by changes in spot luminescence intensity and interparticle distances, as this is related to Debye screening length, which in turn is determined by the charge-to-mass ratio of the particles (i.e., related to size). Therefore, to accurately determine particle sizes and shapes, we selected them from the discharge tube for analysis using a scanning electron microscope (SEM).

**Figure 3:**
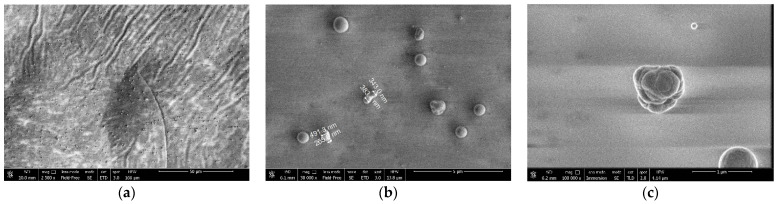
SEM images of nano- and micro-particles: (**a**) nanometer fraction (10–100 nm); (**b**) submicron particles (200–500 nm); (**c**) micron-sized particles.

The synthesized particles were collected on a double-sided carbon tape placed on the discharge glass wall near the lower electrode. After the discharge was switched off, the obtained material was analyzed using SEM. Figure 3 shows the obtained images. As a result of particle synthesis in a stratified DC discharge plasma at room temperature, three distinctive groups of particles of different shapes and sizes were detected. The material contains smooth spherical nanoparticles 10–100 nm in size (Figure 3a). The main group of particles has a very smooth spherical shape and is 200–500 nm in diameter (Figure 3b). There are also dumbbell-like particles of the same size. Finally, micron-sized particles of complex shapes (cauliflower-like), apparently formed by the coagulation of several smaller particles, were also detected (Figure 3c).

**Figure 4:**
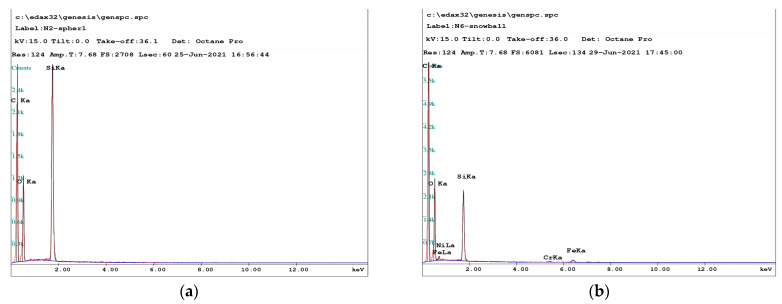
Spectral composition of (**a**) nanoparticles and (**b**) microparticles.

Figure 4 shows the elemental analysis spectra of the synthesized particles. The method is based on energy-dispersive spectral analysis of the samples, performed on an FEI Nova NanoSEM-650 scanning electron microscope. Spectral analysis shows that all particles ranging in size from nanometers to submicrons contain C, O, and Si elements (see Figure 4a). This fact proves that the particles were formed from the sputtered material of the dielectric concentrator made of polydimethylsiloxane (PDMS) [Si(CH_3_)_2_O]*n*. Only large micron-sized particles additionally contain some metal impurities such as Fe, Cr, and Ni (see Figure 4b). Such metal impurities possibly appear due to the sputtering of the stainless steel cathode material. It should be noted that the particles of 1 mcm in size should levitate in the synthesis region for a long time, roughly 10 min, to grow to such a size.

## 3. Discussion

Let us define the main mechanisms leading to the formation of three distinctive groups of particles in a DC glow discharge at room temperature. For this purpose, we will compare the process of particle formation in DC discharge with the results of the known works on particle formation in RF discharges (Section 3.1). Additionally, we will perform the estimations of the gas discharge plasma parameters (Section 3.2) and the particle charges (Section 3.3), as well as the estimation of the forces acting on the particles (Section 3.4). Finally, we will try to analyze the interparticle interaction in terms of the particle coagulation process (Section 3.5).

### 3.1. Comparison of Nanoparticle Growth Processes in DC and RF Discharges

First of all, let us mention the difference in the processes of dust particle formation in gas discharges in pure inert and reactive gases. Different plasma-chemical processes of the gas molecules activation, dissociation, and subsequent coagulation of radicals take place in the bulk of gas discharges in reactive gases [2,3,4,5,7,8,9,10,19,20]. In this case, the particles are formed from the gas dissociation products. Similar dissociation and coagulation processes of gas molecules are absent in the discharges in pure inert gases, but the formation of nano- and microparticles is possible as a result of sputtering of electrodes, substrates, or vacuum chamber walls under the action of electron and ion beams [3,10]. In the present experiments, a sputtering of a dielectric concentrator made of polydimethylsiloxane (PDMS) [Si(CH_3_)_2_O]*n* occurs, leading to the formation of the particles consisting of Si, C, and O elements. Moreover, large particles of 1 mcm in size contain Fe, Cr, and Ni atoms, possibly due to the sputtering of the stainless steel cathode material.

It should be noted that the formation of a bimodal size distribution function of the synthesized particles was first discovered in an RF discharge in SiH4 [4,5]. Since then, this phenomenon has been studied quite well, experiments have been improved, and various models of particle growth based on these experiments have been presented [19]. Bouchoule et al. [4] and Watanabe et al. [5] have suggested that the process of particle growth to a micron size can be divided into three phases: (1) the initial growth phase, (2) the rapid growth phase, and (3) the growth saturation phase. In the initial growth phase, particles of about 10 nm in size appear (begin to be detected by laser and SEM). Particles of this size have a charge number of up to 10. Due to charge fluctuation associated with charge emission from the particle surface and other external influences, the particles can be charged negatively, neutral, or even positively. The first group of small particles remains in the discharge all the time. Their density and size remain virtually unchanged over time. In the rapid growth phase, a second group of particles begins to appear, and their size sharply increases from 10 to 120 nm. In this phase, the charge of small particles (~10 nm) plays a significant role in the process of coagulation into larger particles. It should be noted that two large particles with large charges of the same sign cannot stick together due to Coulomb repulsion between them. Therefore, positively charged particles or neutral particles (molecules, radicals, and even large clusters) can play a significant role in the process of particle growth. Later, the growth saturation phase occurs, when the particle parameters do not change significantly. The authors [5] note that large particles (120 nm) are formed from small ones (10 nm), which is also evident from SEM images of large particles, which have a cauliflower-like shape, i.e., consist of smaller aggregates.

We assume that similar processes occur during the synthesis of nanoparticles in a DC glow discharge. Initially, due to sputtering of the dielectric concentrator, a condensed dispersed phase in the form of a “haze” was observed in the glow of the first stratum above the concentrator. Due to the particles’ small size (10–100 nm), the intensity of laser light reflected from their surface was insufficient, and individual particles were initially indistinguishable. It should be noted that such particles remained in the discharge and were observed in the collected material as smooth spherical particles of 10–100 nm in size (Figure 2a), as it was obtained in RF discharges [4,5]. Several minutes after the start of the plasma generation and sputtering of the concentrator, an increase in the number of particles and their size was observed (Figure 2b,c). The presence of large smooth spherical particles and an increase in their size to diameters of 200–500 nm can be explained by uniform and continuous condensation of neutral or positively charged small nanoparticles on their surfaces. Some particles have a “dumbbell” shape (see Figure 3b), i.e., they were formed by the coagulation of two or more large spherical particles. Finally, as a result of a prolonged synthesis, large micron-sized particles with a cauliflower-like shape were also formed by the coagulation of many smaller particles. It should be noted that further condensation of small nanoparticles on the surface of the dumbbell-like or cauliflower-like particles leads to some smoothing of the particles’ shape.

It should be noted that the first two groups of particles, i.e., smooth spherical and dumbbell-like particles with a size up to 1 mcm, do not contain any metal impurities; thus, no metal elements are involved in their synthesis process. It is these particles that are of interest from the point of view of applicability. The third group of particles, i.e., the largest one of about 1 mcm in size, contains metal impurities on their surfaces and has complex shapes. We can exclude their presence in the material obtained by turning off the discharge earlier.

Let us also note a number of differences between the experiments on the particle synthesis in DC discharge and RF discharges [4,5]. In the present experimental setup with DC discharge, the particles form and grow in the process of gas phase condensation from the sputtered material of the dielectric concentrator, while the gas dissociation products (e.g., different radicals of SiH4) are the main source for the particles in RF discharges. This fact is confirmed by the elemental composition of the particles (see Figure 4a), which shows that the particles mainly consist of C, O, and Si atoms. Finally, three groups of particles significantly different in size and shape were obtained in DC discharge, in contrast to two groups of particles in [4,5].

### 3.2. Gas Discharge Plasma Parameters

The parameters of the synthesized particles are determined to a large extent by the gas discharge conditions. In addition, large, synthesized particles begin to influence the discharge parameters. In many studies [4,5], some plasma instabilities arise in the discharge starting from a certain particle size. Upon reaching a certain size, particles can be periodically carried out of the discharge: either they fall under the action of gravity, or they are pushed away by the flows of ions or neutral particles [7,20]. To analyze the process of particle growth in a gas discharge, it is necessary to evaluate the parameters of the discharge plasma, particles, and the main forces acting on them, which have different dependencies on their radius *r*_0_ (~*r*_0_, ~*r*_0_^2^, ~*r*_0_^3^).

First, let us evaluate the plasma parameters in two discharge regions, i.e., directly at the exit from the dielectric concentrator (the narrowed part of the tube) and in the positive column of the discharge (the normal part of the tube) (see Figure 1). The argon pressure *p* = 14.7 Pa, the voltage of *U* = 3 kV applied to the discharge, and the discharge current of *J* = 2.5 mA were maintained constant in the experiment. The inner diameter of the discharge tube is *D_d_* = 4 cm. The distance between the cathode and anode is 130 cm, the cathode area is equal to the anode area, and *S_el_* = 12.5 cm^2^. The diameter of the exit of the dielectric concentrator is *D_si_* = 0.65 cm.

Let us determine axial electric field strength *E_p_* in the discharge tube from the balance of ionization and ambipolar losses of charged particles to the wall (please see also Supplementary File, Section S3.2). Neglecting nonlocal effects, the radial plasma distribution is determined by the electron and ion balance equations, which are taken in drift-diffusion approximation, supplemented by the Poisson equation for the electric field strength. The electron and ion fluxes in the radial direction are equal to each other in the steady state and, with a weak violation of plasma quasi-neutrality (the electron and ion densities are approximately equal to each other, *n_e_* ≈ *n_i_* = *n*_0_), are determined by the ambipolar diffusion with the diffusion coefficient *D_a_*. To determine the ionization rate constant and electron transport coefficients in argon, the BOLSIG+ program [29,30] was used with the Biagi-v8.9 cross-section set from [31]. To determine the ion mobility, the approximation from [32] was used for the mobility reduced to normal conditions at a temperature of 300 K (*μ_i_* in cm^2^/(V s), *E_p_*/*N* in Td, *N* is the gas density), which was obtained from experimental data in [33,34,35,36,37,38,39]. The resulting values of the reduced field and plasma parameters are presented in Table 1 for the main and the constricted sections of the discharge tube, as well as for two intermediate values of the reduced field. The “radii” *R* of the current cross-sections for these regions were also determined from the condition of ionization balance using the ionization rate and ambipolar diffusion coefficient calculated from the numerical Boltzmann equation for a given value of the reduced field. The resulting radii were used to calculate the plasma density *n*_0_.

Table 1 also presents the mean free paths of electrons *l_e_* and ions *l_i_*, determined from the classical relation connecting the particle diffusion coefficient with their thermal velocity and mean free path. Using the obtained values of the longitudinal electric field strength *E_p_* from the condition of normalization to the discharge current *J*, the electron and ion concentrations on the tube axis *n*_0_ were found (see Table 1). Table 1 also compares the electron drift velocities v*_dr,e_* and ion drift velocities v*_dr,i_* in the positive column of the discharge with their thermal velocities v*th_,e_* and v*th_,i_*. It is seen that in the main part of the discharge tube, the electron drift velocity is two orders of magnitude lower than the thermal one, and in the constricted part, it is an order of magnitude lower. Therefore, the use of the two-term approximation for calculating the electron distribution function (EDF) is still justified. At the same time, in the main part of the tube, the ion drift velocity is comparable to the thermal one, and in the constricted part, it is significantly higher, which must be taken into account when calculating the charge of the particles.

### 3.3. Charges of the Particles

Let us estimate the charges of the particles using the orbital motion limited (OML) theory, which is often used in the field of dusty plasma physics in obtaining a dust particle charge being placed in the gas discharge plasma [40,41]. The OML is applicable in the molecular regime of electron and ion transport, i.e., under the condition that their mean free paths, *l_e_* and *l_i_*, are much greater than the radius of the dust particle *r*_0_, *l_e_* >> *r*_0_, *l_i_* >> *r*_0_. As can be seen from Table 1, this condition is satisfied for micron-sized particles. The equilibrium charge or potential of the surface *ϕ*_0_ of a particle in a plasma is determined by the equality of the electron and ion fluxes onto it. Typically, in gas discharge plasma, a dust particle is charged negatively due to the higher mobility of electrons.

**Figure 5:**
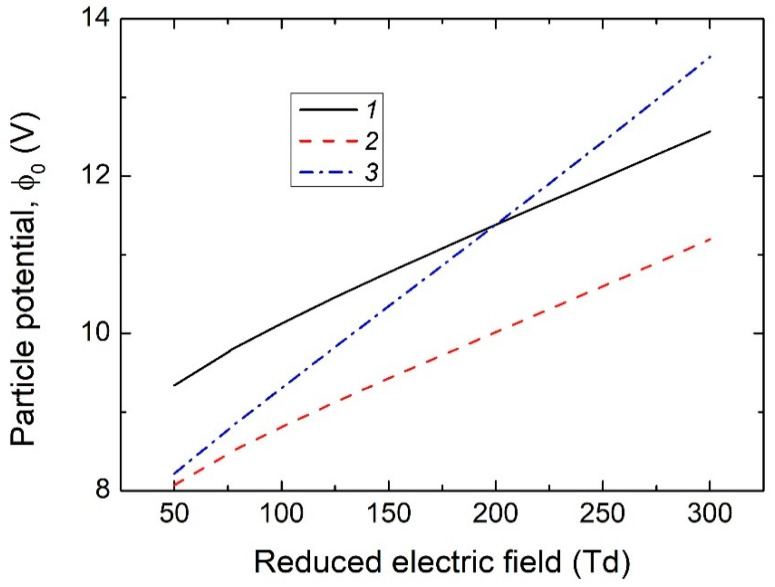
Dependences of the particle surface potential on the reduced electric field calculated with the Maxwellian EDF and IDF (curve *1*), with the Maxwellian IDF and EDF from the numerical solution of the Boltzmann Equation (*2*), and with the shifted Maxwellian IDF and EDF from the numerical solution of the Boltzmann Equation (*3*).

In real plasmas of DC glow discharge or RF discharge, the electron distribution function differs from the Maxwellian form due to the action of an external electric field wall (please see also Supplementary File, Section S3.3). Thus, the solution of the Boltzmann equation for the EDF in two-term approximation was used [42]. For ions, as can be seen from Table 1, the drift velocity in the gas-discharge plasma under study is comparable to the thermal velocity, so the ion distribution function (IDF) is also not Maxwellian, which must be taken into account when calculating the ion flux onto a dust particle. For the estimations, the shifted Maxwellian distribution was used [43,44,45,46,47,48,49]. Figure 5 shows the dependence of the dust particle potential *ϕ*_0_ as a function of the reduced electric field *E_p_*/*N*. In calculations with Maxwell distribution functions for electrons and ions, the ion temperature was assumed to be equal to room temperature, and the electron temperature *T_e_* was determined by the average energy obtained with the EDF from the numerical solution of the Boltzmann equation. It is seen that taking into account the difference between the calculated EDF and the Maxwellian one leads to a decrease in the dust particle potential, while the addition of directed ion motion leads to an increase in the dust particle potential. Moreover, at low *E_p_*/*N*, the particle surface potential is lower than in an equilibrium plasma with two Maxwellian distributions for electrons and ions, and at high *E_p_*/*N*, it is higher.

### 3.4. Forces Acting on the Particles

**Figure 6:**
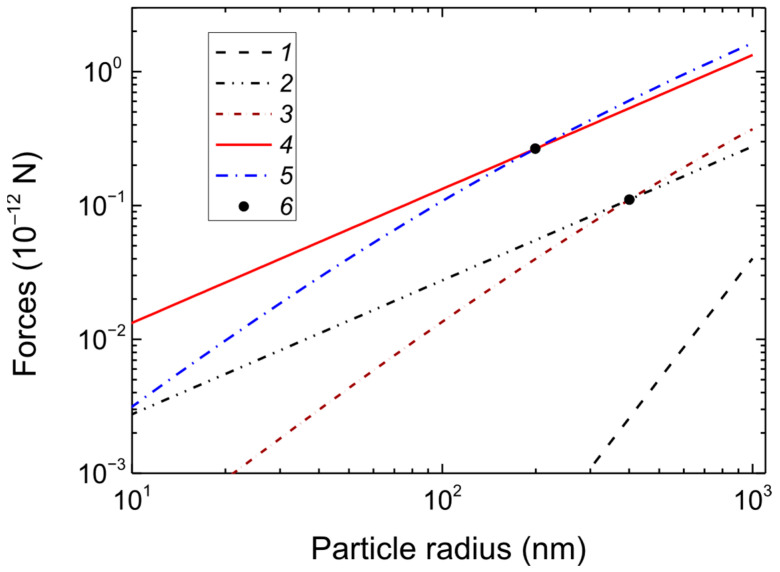
Dependences of the gravity force (*1*), the electric force (*2*,*4*), and the ion drag force (*3*,*5*) in the normal (*2*,*3*) and narrowed parts (*4*,*5*) of the tube on the particle radius. The black circles (*6*) indicate the intersection points of the ion drag force and the electric force.

Now, let us evaluate the forces acting on the particles in the discharge. Typically, the levitation of the dust particles in a stratified DC glow discharge is determined by the balance of the gravitational force *F_g_*, the electrostatic force *F_E_*, and the ion drag force *F_id_* [50,51,52] (please see also Supplementary File, Section S3.4). Note that in a DC discharge in a vertical tube, the ion drag force and the gravitational force are directed downward toward the cathode. Particles levitate due to the electrostatic force acting upward, due to the interaction of the discharge’s electric field with the particle charge. Figure 6 shows the dependences of gravity, electric force, and ion drag force on particle radius *r*_0_ in the normal and constricted sections of the tube. It was assumed that the particles formed in the experiment were polydimethylsiloxane (PDMS) [Si(CH_3_)_2_O]*n,* which has a density of 0.98 at 25 °C. Our estimations of the ion drag force are based on the works [46,47,48,49] (and the literature cited therein), where the ion drag force consists of two parts, i.e., due to ions absorbed by dust particles and the contribution of scattered ions. Gravity has the strongest dependence on radius ~*r*_0_^3^. Therefore, as particle size increases beyond a micron, the probability of particles falling to the bottom of the discharge increases sharply. However, for particles with a radius less than 1 μm, gravity can be neglected in both the normal and narrowed parts of the discharge tube. In the normal part of the discharge, with a reduced electric field of *E_p_*/*N* = 77.4 Td, the surface potential of the macroparticles is *ϕ*_0_ = 8.823 V, while in the narrowed part, with *E_p_*/*N* = 258.3 Td, it is *ϕ*_0_ = 12.65 V (see Table 1 and Figure 1). Therefore, the electrostatic force in the narrowed part of the discharge, where both the particle charge and the field strength are higher, is significantly greater than in the normal part.

**Figure 7:**
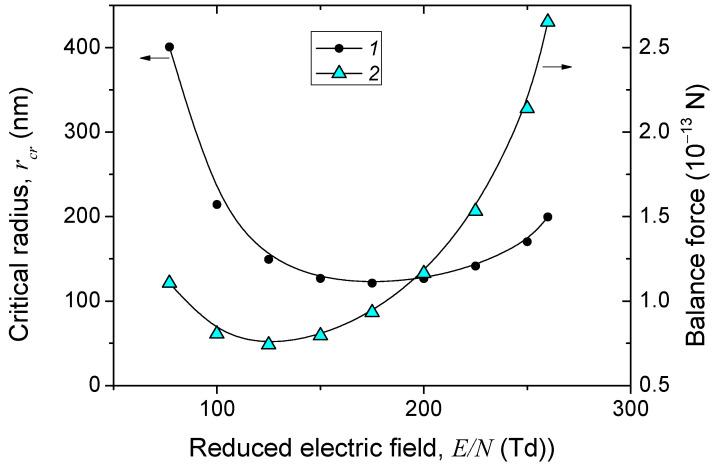
Dependences on the reduced electric field of the critical radius of particles (curve *1*) and the electric force and ion drag force equal to each other at this particle radius (curve *2*) (see also Supplementary File, Figure S1).

The critical radius *r_cr_* of the particles, which determines the upper limit of the radius of dust particles that can levitate in the region at a given value of the reduced field, is presented in Figure 7. It is seen that the critical radius varies nonmonotonically with *E_p_*/*N*. Particles with radii between 120 and 200 nm can levitate above the discharge constriction region, while particles with smaller radii will float upward into the main discharge region, since the electrical force is greater than the ion drag force. It should be noted that the formation of a dust particle cloud will not change the electrical force if the particle charge remains constant, but the ion drag force can significantly weaken, leading to an increase in the critical radius of the dust particles.

The presence of two other forces acting on the particles in an upward direction should be noted, i.e., the thermophoretic force and the neutral drag force. It is known that a gas temperature difference of just a few degrees per centimeter can lead to the levitation of particles in the discharge. Indeed, the gas above the dielectric concentrator sputtered by the ion bombardment can be significantly heated, and the temperature difference in the region of particle levitation can be valuable. There is no induced gas flow in the experiment, but the sputtered products of the dielectric concentrator could affect the levitated particles, pushing them up. Though there is no necessary data for the evaluation of these forces, they seem to be negligible under our conditions compared to previously described forces.

### 3.5. Interaction of Charged Particles

In order to explain the mechanisms of the formation of large dumbbell- or cauliflower-shaped particles resulting from the coagulation of smaller particles, we carried out the following analysis (please, see also Supplementary File, Section S3.5). Let us consider the shielded electrostatic and van der Waals interactions between two particles [53,54,55]. We have analyzed the dependences of the total interaction energy, which includes the potential of shielded electrostatic interaction of charged particles and the van der Waals interaction energy, on the smallest distance between the particle surfaces. The particles were assumed to have a spherical shape and were made of polydimethylsiloxane, which is the main silicone compound. Polydimethylsiloxane is a linear polymer of dimethylsiloxane, which is often called silicone in everyday use. The number of dimethylsiloxane units in the structure can reach 15,000. Depending on the length of the polymer chain, substances with different physical properties are obtained. The viscosity of such compounds increases with increasing length, which corresponds to the transition from very mobile, gasoline-like liquids to more viscous silicone oils and, finally, to resinous substances. Thus, the small particles can be in a liquid state, which may explain the spherical shape of the synthesized nanoparticles. As their size increases, the PDMS particles become more solid. In the calculations of the van der Waals force, the parameters were used for PDMS [56,57,58,59]. The method for calculating the van der Waals interaction and screened electrostatic interaction is described in detail in [55].

The calculations were performed for two cases, i.e., the interaction of one 10 nm particle with another particle of size in the range 10–500 nm and the interaction of one 500 nm particle with the particles of different sizes (10–500 nm). The results obtained for a 10 nm particle at constant particle charge reveal that the energy reduced to the charges decreases with increasing particle radius, but the total energy remains significantly higher than the thermal energy (see Supplementary File, Figure S2). When particles approach each other at constant potential, the total energy decreases but still remains significantly higher than the thermal energy. A similar picture holds for the interaction of the 500 nm particle with particles of different radii (see Supplementary File, Figure S3). Consequently, thermal coagulation of particles with radii of 10 nm and above will be strongly suppressed by electrostatic repulsion. Note that the small contribution of van der Waals interactions to the total energy and the absence of electrostatic attraction at short distances are due to the low permittivity of silicone. As noted above, large particles can contain metal atoms. At sufficiently high quantities, this can lead to a significant increase in the effective permittivity and the emergence of an attractive force between particles at short distances due to both electrostatic and van der Waals interactions.

Above, we have considered the static interaction of a pair of charged particles. Let us analyze the situation when the particles are approaching each other at some starting velocity. Indeed, the synthesized particles located above the concentrator (see Figure 2) exhibit various forms of motion, from chaotic thermal motion to pronounced directed motion of individual particles and even collective vortex or wave motion. Numerous studies on the propagation of nonlinear waves in plasma-dust structures in a DC glow discharge have experimentally and theoretically demonstrated the possibility of particle transport along the structure (see, for example, [60]). The experimental measurements [61] at similar DC discharge conditions show that the particles’ relative velocity could achieve the value of 6 cm/sec. Particles move towards each other when the moving dust component runs onto the stationary fraction in the vertical waves. Simple estimates indicate that for particles 200 nm in size and larger, with an initial relative velocity of 4 cm/sec or higher, the energy directed to the other motion of two particles may be sufficient to overcome the screened repulsive potential. Taking into account various additional stochastic processes, this fact may explain the presence of large dumbbell- or cauliflower-shaped particles resulting from the coagulation of smaller particles.

## 4. Conclusions

The paper presents a novel method for the synthesis of nanoparticles of different sizes, compositions, and shapes in a stratified DC glow discharge. Time evolution of the particle growth, SEM images of the collected material, as well as its elemental analysis using EDX, were presented and analyzed.

Three distinctive groups of particles were found: smooth spherical nanoparticles 10–100 nm in size, smooth spherical particles 200–500 nm in size, and their dumbbell-shaped agglomerates, and large micron-sized cauliflower-shaped particles. Elemental analysis revealed that all particles are formed from the material of the sputtered dielectric concentrator made of polydimethylsiloxane, and the third group of micron-sized cauliflower-shaped particles additionally contains some metal impurities coming from the sputtered stainless steel electrode. The small particles of the first group are produced in the process of condensation of sputtered material in the gas phase and levitate in the synthesis region throughout the entire duration of the experiment. The presence of large smooth spherical nanoparticles and an increase in their size to diameters of 200–500 nm can be explained by uniform and continuous condensation of neutral or positively charged small nanoparticles on their surfaces. Dumbbell-like particles are formed in the process of coagulation of two or more large spherical particles. Finally, as a result of a prolonged synthesis, large micron-sized particles with a cauliflower-like shape are also formed by the coagulation of many smaller particles.

Analysis of the discharge plasma parameters, particle charges, and the forces acting on the particles showed that nanoparticle synthesis in DC discharge with a dielectric concentrator is determined mainly by the balance of electrostatic and ion drag forces. Analysis of screened electrostatic repulsion and van der Waals interactions between two particles showed that thermal coagulation of particles with radii of 10 nm and above will be strongly suppressed by electrostatic repulsion. However, the presence of a relative velocity greater than 4 cm/s between two particles larger than 200 nm may be sufficient for their collision, which may explain the presence of large dumbbell- or cauliflower-shaped particles.

Synthesized smooth spherical particles consisting of silicon, carbon, and oxygen can be used as catalysts, absorbents for purifying gases and liquids, in composite materials to improve their mechanical properties, and as carriers for drug delivery. The presented method of nanoparticles synthesis can be used for the production of nanoparticles of different elemental composition by varying the dielectric concentrator material. A specific fraction of nanoparticles by size can be selected by varying the discharge parameters and the time of the discharge burning.

## Figures and Tables

**Table 1:** Plasma parameters in the normal, transition, and narrowed parts of the discharge tube.

Parameter	Normal Part of the Tube	Transition Part 1	Transition Part 2	Narrowed Part of the Tube
*R*, cm	2.000	1.31	0.70	0.325
*E_p_*/*N* (Td)	77	100	150	260
*E_p_* (V/cm)	2.81	3.64	5.45	9.45
*T_e_* (eV)	4.402	4.576	4.926	5.65
*n*_0_ (cm^−3^)	4.7 × 10^8^	9.02 × 10^8^	2.24 × 10^9^	6.6 × 10^9^
*ϕ*_0_ (V)	8.823	9.302	10.34	12.65
*l_e_* (cm)	0.40	0.38	0.36	0.33
*l_i_* (cm)	1.82 × 10^−2^	1.74 × 10^−2^	1.58 × 10^−2^	1.34 × 10^−2^
v*th_,e_* (cm/s)	1.40 × 10^8^	1.43 × 10^8^	1.48 × 10^8^	1.59 × 10^8^
v*_dr,e_* (cm/s)	6.00 × 10^6^	7.43 × 10^6^	1.05 × 10^7^	1.68 × 10^7^
v*th_,i_* (cm/s)	3.98 × 10^4^	3.98 × 10^4^	3.98 × 10^4^	3.98 × 10^4^
v*_dr,i_* (cm/s)	2.64 × 10^4^	3.25 × 10^4^	4.44 × 10^4^	6.51 × 10^4^

## Data Availability

Data is contained within the article or Supplementary Material.

## Supplementary Materials

The supporting information can be downloaded at: https://www.mdpi.com/article/10.3390/nano15231802/s1, Figure S1: Electrostatic force (solid curves) and ion drag force (dashed curves) acting on particles, Figure S2: The total interaction energy reduced to charges of two particles with ra dii a1 = 10 nm and a2 = 10 (*1*,*6*), 20 (*2*), 50 (*3*), 100 (*4*), and 500 nm (*5*,*7*) as a function of the smallest distance between their surfaces with potential *Φ*_0_ = 8.823 V at *R* = ∞. Curves (*1*–*5*) for constant charges, (*6*,*7*) for constant surface potentials, Figure S3: The total interaction energy reduced to charges of two particles with radii a1 = 500 nm and a2 = 500 (*1*,*6*), 350 (*2*), 200 (*3*), 50 (*4*), and 10 nm (*5*,*7*) as a function of the smallest distance between their surfaces with potential *Φ*_0_ = 8.823 V at *R* = ∞. Curves (*1*–*5*) for constant charges, (*6*,*7*) for constant surface potentials, Figure S4: The total interaction energy of two particles with radii a1 = 10 nm and a2 = 10 (*1*,*3*) and 500 nm (*2*,*4*) as a function of the smallest distance between their surfaces with potential *Φ*_0_ = 8.823 V at *R* = ∞. Curves (*1*,*2*) for constant charges, (*3*,*4*) for constant surface potentials. The line *k_B_T* is the thermal energy of particles at *T* = 300 K.
