# Supplementary material for: Synthesis of Nanoparticles of Different Morphology in a DC Discharge
Source: Nanomaterials (Basel). 2025 Nov 29;15(23):1802. doi: 10.3390/nano15231802 (PMC12692907; doi:10.3390/nano15231802)
Supplement: Supplementary file 1 [file nanomaterials-15-01802-s001.zip › nanomaterials-3993416-supplementary.pdf]

Supplementary Materials

for

Synthesis of nanoparticles of different morphology in a DC discharge

A.V. Fedoseev[1], A.V. Filippov[1], M.M. Vasiliev[1], O.F. Petrov[1]

[1]Joint Institute for High Temperatures RAS, Moscow, Russia

Corresponding author: Alexander Fedoseev, alex.fed245@gmail.com

Section S3.2. Gas discharge plasma parameters

At the wall of the gas-discharge tube, we use zero boundary conditions for electrons and ions (the condition of no emission from the walls of the gas-discharge tube), which can be replaced by effective boundary conditions to obtain the correct flux of charged particles to the walls, i.e. the correct loss rate of charged particles. As a result, we obtain the well-known Schottky solution for the plasma density distribution and an implicit equation for reduced electric field strength:

$$\nu_{ion}\left(E/N\right)=\left(\frac{\lambda_{0,1}}{R}\right)^{2}D_{a}\left(E/N\right), \qquad \text{(SM.1)}$$

where $\lambda_{0,1}\approx 2.4048$ is the first root of the Bessel function $J_0(x)$, R is the discharge tube radius, $\nu_{\text{ion}} = k_{\text{ion}}N$ is the gas ionization frequency, k_{ion} is gas ionization rate constant, N is gas atom density, E is electric field strength. The ambipolar diffusion coefficient D_a can be determined as:

$$D_a=\frac{\mu_e D_i+\mu_i D_e}{\mu_e+\mu_i},$$

where D_e, D_i, μ_e, and $\mu_i$are diffusion and mobility coefficients of electrons and ions, respectively. In equation (SM.1), the ionization rate constant is the sharpest function of the reduced field E/N, and the transport coefficients depend on E/N much lesser.

Section S3.3. Charges of the particles

Under typical DC glow discharge conditions, the EDF is weakly anisotropic in velocity. Therefore, a two-term approximation is applicable to its description, where only the first two terms are considered in the expansion of the distribution function in spherical harmonics. After integration over the angular variables, the contribution of the dipole term to the EDF vanishes, and for the electron flux density to the particle, we obtain the expression [39]:

$$J_e\left(\phi_0\right)=\sqrt{\frac{2}{m_e}}\int_0^{\infty} f_{0e}\left(\varepsilon\right)\sigma_e\left(\varepsilon,\phi_0\right)\varepsilon d\varepsilon, \qquad \text{(SM.2)}$$

where ϕ_0 is electric potential of the dust particle surface, σ_e is the cross-section of electron absorption by the dust particle, f_{0e} is the spherically symmetric part of the EDF, m_e and ε is the electron mass and energy.

Using the shifted Maxwellian distribution for IDF, the following expression can be obtained for the ion flux [40-46]:

$$J_i\left(\phi_0, v_{i,dr}\right) = n_{i0} v_{th,i} \pi r_0^2 \left[\left(1 - 2\frac{e\phi_0}{T_i} + 2x^2\right)\frac{\sqrt{\pi}}{4x}\operatorname{erf}\left(x\right) + \frac{1}{2}\exp\left(-x^2\right)\right] \tag{SM.3}$$

where the dimensionless quantity x is defined by the expressions:

$$v_{th.i} = \sqrt{\frac{8T_i}{\pi m_i}}, \quad x = \frac{2}{\sqrt{\pi}}\frac{v_{i,dr}}{v_{th,i}}, \tag{SM.4}$$

where T_i is the ion temperature in energy units, m_i is the ion mass. With the Maxwellian EDF, the well-known expression for the electron flux density follows from (SM.2) [38]. At low velocities of directed ion motion, using the behavior of the error function and the exponent for small values of the argument (for $x << 1$ function $\operatorname{erf}(x) \approx 2x/\pi^{1/2}$), the standard expression for the ion flux density also follows from (SM.3) [38].

Next, by numerically integration of (SM.2) and using the condition of equality of electron and ion fluxes (SM.2 and SM.3) in the steady state, we find the dust particle electric potential using the bisection method.

Section S3.4. Forces acting on the particles

The ion drag force is calculated based on the work [45] (see also the works [43,44,46] and the literature cited therein). The ion drag force F_{id} consists of two parts, $F_{id} = F_c + F_{sc}$, which are described by the expressions:

$$F_c = \frac{\sqrt{\pi} n_i r_0^2 T_i}{x^2}\left\{x\left(1 + 2x^2 - \frac{2e\phi_0}{T_i}\right)e^{-x^2} + \left[4x^4 + 4x^2 - 1 + 2\left(1 - 2x^2\right)\frac{e\phi_0}{T_i}\right]\frac{\sqrt{\pi}}{2}\operatorname{erf}\left(x\right)\right\}, \tag{SM.5}$$

$$F_{sc} = 4\pi n_i r_0^2 \frac{e^2\phi_0^2}{T_i} G\left(x\right)\ln\Lambda, \tag{SM.6}$$

where F_c is force due to ions absorbed by dust particles and F_{sc} is the contribution of scattered ions, G is Chandrasekhar function:

$$G\left(x\right) = \frac{1}{x^2\sqrt{\pi}}\left[\frac{\sqrt{\pi}}{2}\operatorname{erf}\left(x\right) - xe^{-x^2}\right], \tag{SM.7}$$

$\ln\Lambda$ is Coulomb logarithm:

$$\ln\Lambda = \ln\left(\frac{R_s + b_{\pi/2}}{b_{\pi/2} + r_0}\right) = \ln\left[\frac{1 + k_s b_{\pi/2}}{k_s\left(b_{\pi/2} + r_0\right)}\right], \tag{SM.8}$$

where k_s and R_s are the shielding constant and radius, $b_{\pi/2}$ is impact parameter of ion scattering at an angle π/2 (Landau radius). These quantities are defined by the relations ($R_s = 1/k_s$):

$$b_{\pi/2} = \frac{e\phi_0}{2T_i + m_i v_{i,dr}^2} r_0,\ k_s^{-2} = r_0^2 + \left(k_{De}^2 + k_{Di}^2\right)^{-1}, k_{Di}^2 = \frac{4\pi n_i e^2}{T_i + m_i v_{i,dr}^2}. \tag{SM.9}$$

Note that the difference between the calculated EDF and the Maxwellian one also leads to a change in the electron screening constant [39]:

$$k_{De}^2 = \frac{4\pi e^2 n_e}{T_e}\zeta_e, \tag{SM.10}$$

где ζ_e is factor which takes into account the difference between the calculated EDF and the Maxwellian one:

$$\zeta_e = -T_e \int_0^\infty \frac{\partial f_{0e}(\varepsilon)}{\partial \varepsilon}\sqrt{\varepsilon}d\varepsilon. \tag{SM.11}$$

For the Maxwellian EDF $\zeta_e = 1$.

In [45], the following expressions were also proposed to determine the screening radius and the Landau radius:

$$b_{\pi/2} = r_0 \frac{e\phi_0}{m_i v_{eff}^2},\ R_s^2 = r_0^2 + \left[\left(k_{De}^2 + k_{Di,0}^2 \frac{2T_i}{m_i v_{eff}^2}\right)\right]^{-1}, \tag{SM.12}$$

where v_{eff} is the effective ion velocity, which in [45] is defined as follows:

$$m_i v_{eff}^2 = 2T_i + m_i v_{dr,i}^2 \left\{1 + \left[\frac{v_{dr.i}\sqrt{m_i/T_e}}{0.6 + 0.05\ln\left(m_i/m_p\right) + \left(R_{De}/5r_0\right)\left(\sqrt{T_i/T_e} - 0.1\right)}\right]^3\right\}. \tag{SM.13}$$

Here m_p is the mass of proton.

The following expressions for other forces were used [48].

Gravity force $\boldsymbol{F}_g$:

$$\boldsymbol{F}_g = m_d \boldsymbol{g}, \tag{SM.14}$$

where $\boldsymbol{g}$ is the gravitational acceleration and $m_d \sim r_0^3$ is the particle mass.

Electrostatic force $\boldsymbol{F}_E$:

$$\boldsymbol{F}_E = eZ_d\boldsymbol{E} \tag{SM.15}$$

where $\boldsymbol{E}$ is the electric field strength, Z_d is the dust particle charge number.

Neutral drag force $\boldsymbol{F}_n$:

$$\boldsymbol{F}_n = -m_d \nu_{dn} \boldsymbol{u}, \tag{SM.16}$$

where ν_{dn} is the effective momentum transfer frequency in collisions of neutral particles with dust particle, $\boldsymbol{u}$ is the speed of dust particle relative to the gas.

Thermophoretic force F_{th}:

$$\vec{F}_{th} \approx -\frac{16}{9}\frac{r_0^2}{\sigma_{tr}}\vec{\nabla}T_n, \tag{SM.17}$$

where σ_{tr} is transport scattering cross-section for gas atoms or molecules, ∇T is the gas temperature gradient.

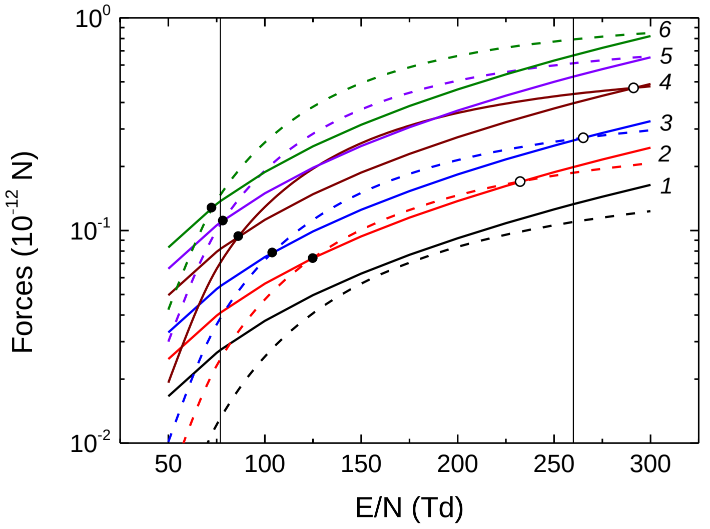

Figure S1. Electrostatic force (solid curves) and ion drag force (dashed curves) acting on particles with radii of 100 nm (*1*), 150 nm (*2*), 200 nm (*3*), 300 nm (*4*), 400 nm (*5*), and 500 nm (*6*). The open and closed circles indicate the *E*/*N* values at which these forces intersect. The vertical lines indicate the *E*/*N* values in the normal and constricted portions of the tube. For particles with a radius of 100 nm, there is no intersection of forces in the studied range of the *E*/*N* parameter. However, the electric force for particles of this radius throughout the discharge exceeds the ion drag force, so they will float upward. The equilibrium positions marked with open circles are points of stable equilibrium, while those marked with closed circles are points of unstable equilibrium, since, when a particle is shifted to the left or right, the force removing the particle from this position begins to predominate.

Section S3.5. Interaction of charged particles

The electrostatic shielded and van der Waals interactions of two particles with each other are estimated based on the works [50-52]. In the calculations of the van der Waals force, the following parameters were used [53-56]. For Ar $\lambda_0 = 106.66$ nm, $n_{02} = 1.00$, $\varepsilon_{20} = 1.0$, $\omega_{UV2} = 1.767 \times 10^{16}$ c^{-1};

for PDMS n_{01} = 1.4, the real part of the permittivity ε_{10} = 2.77, the ultraviolet absorption limit was assumed as for Polystyrene $\omega_{UV1} = 1.432\times10^{16}$ s^{-1}. In the calculations of the screened electrostatic interaction at constant charges, it was assumed that the charges of the particles are related to their radius by the ratio: $q_i = \phi_0 a_i(1+k_D a_i)$, i=1,2. Here, a_i are the particle radii and k_D is the screening constant, taking into account the difference of the EDF from the Maxwellian one. In the case of constant particle surface potentials, their charge depends on the distance between them. Therefore, to convert the interaction energy to unit charges, the charge values at $R = \infty$ were used (which are designated as q_{10} and q_{20} and are equal to the particle charges in the case of constant charges). The method for calculating the van der Waals interaction and screened electrostatic interaction is described in detail in [52]. Figure SM.2 shows the dependence of the total interaction energy of a 10 nm particle on the interparticle distance with the particles of different sizes. It is seen that, at constant charges, the energy reduced to the charges decreases with increasing particle radius, but the total energy remains significantly higher than the thermal energy. When particles approach each other at constant potentials, the total energy decreases but still remains significantly higher than the thermal energy. A similar picture holds for the interaction of a 500 nm particle with particles of different radii (See Fig. SM.3). Consequently, thermal coagulation of particles with radii of 10 nm and above will be strongly suppressed by electrostatic repulsion. Note that the small contribution of van der Waals interactions to the total energy and the absence of electrostatic attraction at short distances are due to the low permittivity of silicone.

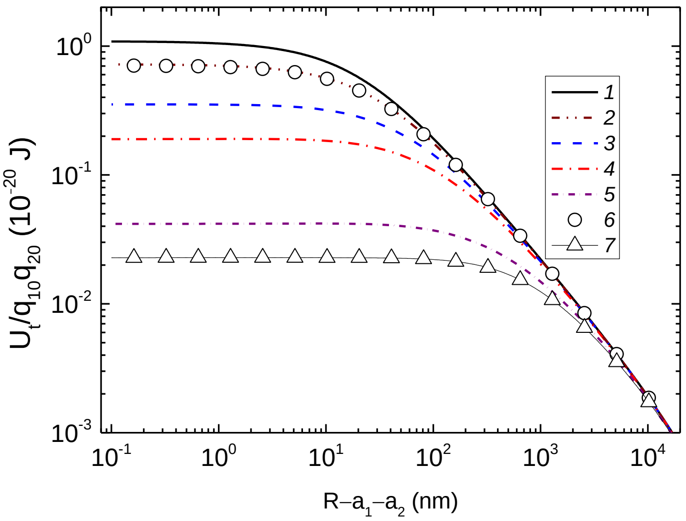

Figure S2. The total interaction energy reduced to charges of two particles with ra dii a_1 = 10 nm and a_2 = 10 (*1,6*), 20 (*2*), 50 (*3*), 100 (*4*), and 500 nm (*5,7*) as a function of the smallest distance between their surfaces with potential ϕ_0 = 8.823 V at $R = \infty$. Curves (*1-5*) for constant charges, (*6,7*) for constant surface potentials.

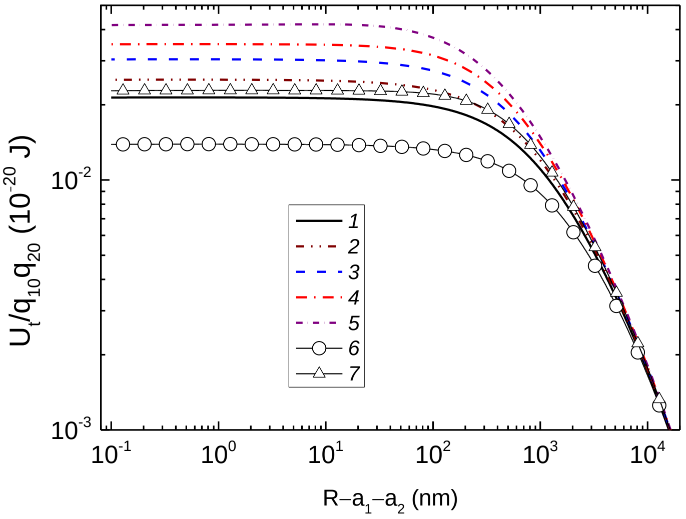

Figure S3. The total interaction energy reduced to charges of two particles with radii a_1 = 500 nm and a_2 = 500 (*1*,*6*), 350 (*2*), 200 (*3*), 50 (*4*), and 10 nm (*5*,*7*) as a function of the smallest distance between their surfaces with potential ϕ_0 = 8.823 V at $R = \infty$. Curves (*1-5*) for constant charges, (*6*,*7*) for constant surface potentials.

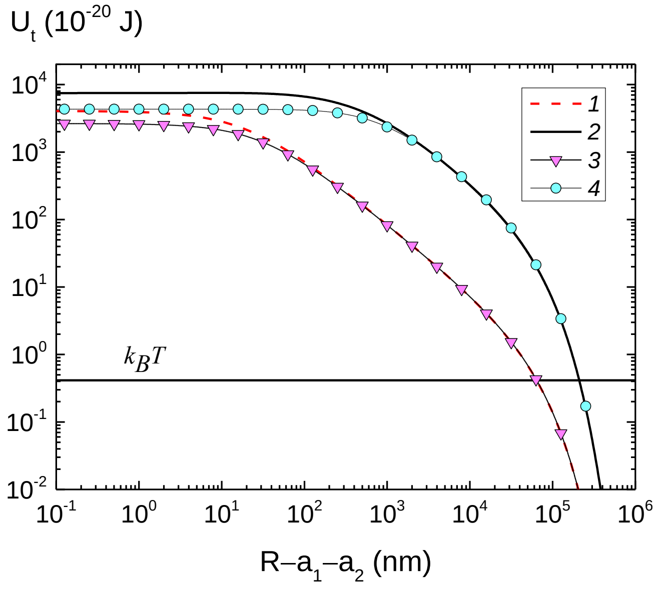

Figure S4. The total interaction energy of two particles with radii a_1 = 10 nm and a_2 = 10 (*1*,*3*) and 500 nm (*2*,*4*) as a function of the smallest distance between their surfaces with potential ϕ_0 = 8.823 V at $R = \infty$. Curves (*1,2*) for constant charges, (*3*,*4*) for constant surface potentials. The line $k_B T$ is the thermal energy of particles at T = 300 K.

At short distances, the interaction energy is significantly greater than the thermal energy. At longer distances, the interaction tends to the DLVO-potential, and the difference between the cases of constant charges and surface potentials disappears.
